# Supplementary material for: Group decisions based on confidence weighted majority voting
Source: Cogn Res Princ Implic. 2021 Mar 15;6:18. doi: 10.1186/s41235-021-00279-0 (PMC7960862; doi:10.1186/s41235-021-00279-0)
Supplement: Supplementary file 1 — Additional file 1: Summary statistics and parameter estimates for our experiment. For each group, we report the average accuracy (%-correct responses across 12 trials) for real and simulated groups, regression coefficients a and b (real confidence = a + b · ideal confidence + ε), and fitted parameters from our formal cognitive modeling approach: equality effect β, group confidence effect γ and group precision σg. Additionally, we report mean, standard deviation as well as the more robust median and quartiles. [file 41235_2021_279_MOESM1_ESM.pdf]

Table 1 Summary statistics and parameter estimates. For each group, we report the average accuracy (%-correct responses across 12 trials) for real and simulated groups, regression coefficients $\boldsymbol{a}$ and $\boldsymbol{b}$ (real confidence $= \boldsymbol{a} + \boldsymbol{b} \times$ ideal confidence $+ \boldsymbol{\epsilon}$), and fitted parameters from our formal cognitive modeling approach: equality bias $\boldsymbol{\beta}$, group confidence bias $\boldsymbol{\gamma}$ and group precision $\boldsymbol{\sigma_g}$. Additionally, we report mean, standard deviation as well as the more robust median and quartiles.

Group	Correct Predictions [%]			Regression		Formal Cognitive Model		
	Real	CWMV	MV	Intercept a	Slope b	β	γ	σ_g
1	75.0	75.0	66.7	43.50	0.94	0.68	0.62	6.0
2	75.0	83.3	75.0	49.46	0.70	1.23	0.51	8.0
3	58.3	83.3	58.3	37.44	0.77	0.53	0.38	10.0
4	83.3	66.7	66.7	58.39	0.35	0.80	0.52	11.0
5	75.0	75.0	75.0	41.44	0.99	0.23	0.46	13.0
6	83.3	83.3	75.0	51.77	1.00	0.62	0.61	7.0
7	83.3	66.7	50.0	48.62	0.76	0.57	0.59	17.0
Mean	76.2	76.2	66.7	47.23	0.79	0.67	0.53	10.9
SD	8.9	7.5	9.6	7.02	0.23	0.30	0.09	3.8
Median	75.0	75.0	66.7	48.62	0.77	0.62	0.52	10.0
25%-Quantile	75.0	70.8	62.5	42.47	0.73	0.55	0.48	7.5
75%-Quantile	83.3	83.3	75.0	50.61	0.96	0.74	0.60	12.0
